# The As and Bs of titi monkey linguistics: why emotional communication is not the enemy

**DOI:** 10.1002/brv.70131

**Published:** 2026-01-20

**Authors:** Mélissa Berthet, Geoffrey Mesbahi, Maël Leroux

**Affiliations:** ^1^ Centre d’Etude en Ethologie et Cognition (CEEC) Univ Rennes, Normandie Univ, CNRS UMR 6552 Rennes F‐35000 France; ^2^ Department of Livestock Sciences Research Institute of Organic Agriculture FiBL Frick 5070 Switzerland

**Keywords:** animal linguistics, syntax, compositionality, semantics, pragmatics, arousal, reasoning, inference, evolution of language

## Abstract

The alarm call sequences of titi monkeys (genera *Plecturocebus, Callicebus* and *Cheracebus*) have sparked important debates over whether they exhibit parallels with human language. Some researchers consider these sequences to involve both semantics and syntax, while others argue that the sequences convey semantic information without syntax. In this review, we revisit this issue by applying fine‐grained linguistic analyses to the most comprehensive data set of titi monkey alarm sequences available to date. Specifically, we evaluate three competing hypotheses: one rich hypothesis suggesting that titi alarm sequences are compositional, and two deflationary alternatives. The first deflationary hypothesis holds that an alarm sequence is a single vocalisation that only superficially resembles a combination of discrete calls. The second deflationary hypothesis posits that an alarm sequence consists of a series of independent calls emitted in rapid succession, governed by no syntactic rule. The data set allows us to reject the first deflationary hypothesis but not the second, preventing us from concluding that titi monkey sequences are compositional. This leads us to another important question: if the sequences are not compositional, what information do they convey, and how? We examine the information encoded in the alarm calls and find that alarm calls likely reflect the caller's arousal level at the time of emission of the call: A‐calls encode high arousal, B‐calls lower arousal, and sequences appear to track dynamic changes in arousal over time. However, strikingly, receivers still manage to extract relevant information about the event eliciting alarm sequences, likely through inferences relying on contextual cues and prior knowledge. This pragmatic enrichment suggests that emotion‐based communication can give rise to complex cognitive processes, particularly on the receiver's side. Titi monkeys thus offer a valuable model for investigating the evolutionary roots of pragmatics. More broadly, our review challenges the misconception that emotional communication is cognitively simple, and invites renewed attention to the role of affective communication in the emergence of linguistic‐like abilities.

## I. INTRODUCTION

Investigating the parallels between human language and animal communication serves two main agendas. For some, it helps understand how and when human language emerged (Hauser, Chomsky & Fitch, 2002). For others, it helps unveil the diversity of communication systems in non‐human animals and retrace their local evolution (Schlenker *et al*., 2016a, 2022; Fischer, 2017). These studies often focus on three core properties of human language: semantics, pragmatics, and syntax [see Berthet *et al*. (2023) for a review]. Semantics (or ‘semantic denotation’) pertains to the meaning of the signal that remains stable across contexts while pragmatics pertains to aspects of meaning that depend on the context. For example, the sentence ‘It is raining outside’ has one semantic denotation, that water is falling from the sky, regardless of its context of emission. However, saying ‘It is raining outside’ to someone about to go to work further means ‘Don't forget to take your umbrella’, while saying it to someone about to take the laundry out also means ‘Hang the laundry inside’. These are examples of pragmatic inferences, where listeners derive additional meaning based on contextual cues. Finally, syntax describes the set of rules that determines whether a sentence is well formed: it allows combination and ordering of elements. In human language, compositional syntax, i.e. the capacity to combine meaningful elements into larger structures whose meaning is derived from the meaning of its elements and the way they are organised, is the most striking form of syntax as it is responsible for language's infinite generative power. An example of compositional syntax is the phrase ‘blue dress’, whose meaning is derived from the order and the meaning of the words ‘blue’ and ‘dress’. This is different from non‐compositional syntax, like idiomatic expressions whose meaning is not derived from the meaning of the words (e.g. ‘it is raining cats and dogs’ means that it is raining strongly, which does not derive from ‘cats’ and ‘dogs’).

Alarm vocalisations are good candidates to study linguistic‐like capacities in non‐human species because (*i*) they can be distinguished easily from other vocalisations in the repertoire, (*ii*) the situations that elicit them are easy to identify, and (*iii*) the behavioural reactions of receivers are conspicuous (Macedonia & Evans, 1993; Zuberbühler, 2009). Building on the pioneering research of Seyfarth, Cheney & Marler (1980, 1980) on the vervet monkey (*Cercopithecus aethiops*) alarm call system, research across various taxa has investigated these calls for referentiality (Crockford *et al*., 2012), syntax (Engesser, Ridley & Townsend, 2016; Suzuki & Zuberbühler, 2019; Leroux *et al*., 2023), deception (Fujita, Kuroshima & Masuda, 2002), vocal displacement (Lameira & Call, 2018) and other language‐like capacities (see Berthet *et al*., 2023; León, Berthet & Zuberbühler, 2024).

Among primates, alarm sequences of titi monkeys (genera *Plecturocebus*, *Callicebus* and *Cheracebus*; Byrne *et al*., 2016) have been argued to exhibit striking parallels with human language, sparking interesting debates among biologists and linguists. Whilst the former argued that titi monkey alarm sequences involve both semantics and syntax (e.g. Cäsar *et al*., 2013), the latter contend that they involve semantics but not syntax (e.g. Schlenker *et al*., 2017). In this review, we aim to settle this debate using the most up‐to‐date data set available on titi monkey alarm sequences, combined with fine‐grained linguistic analyses.

Titi monkeys are American primates. Their small body size and strict arboreality make them subject to high predation pressure, particularly from birds of prey (Ferrari, 2009). They also descend occasionally to the ground, mostly to forage (Souza‐Alves *et al*., 2019), which increases their exposure to terrestrial predators like mammalian carnivores (felids and tayra *Eira barbara*) and snakes (Mourthé *et al*., 2007; Cäsar, 2011; Bicca‐Marques & Heymann, 2013).

Black‐fronted titi monkeys *Callicebus nigrifrons* produce two soft alarm calls in response to threats: the A‐call and the B‐call (Cäsar *et al*., 2012a). A‐ and B‐calls can be emitted either alone (one single call) or combined in long sequences composed of A‐calls, B‐calls, or mixed sequences. If alarm sequences continue, A‐ and B‐calls are gradually replaced by medium and loud calls (Cäsar, 2011), perhaps to deter the predator or signal its presence to neighbouring groups.

In the following sections, we first review previous hypotheses on the syntax and semantics of titi monkey alarm sequences (Section II). We next describe the data set used in our analyses (Section III) and apply a linguistic framework to test competing syntactic interpretations (Section IV). We then explore the semantics (Section V) and pragmatics (Section VI) of the A‐ and B‐calls, and finally discuss the broader implications of our findings for the evolution of language (Section VII).

## II. PREVIOUS HYPOTHESES

Following a series of playback and predator presentation experiments on wild subjects, Cäsar *et al*. (2012b, 2013) argued that titi monkey alarm call sequences represented a sophisticated syntax/semantic interface. They identified four types of alarm sequences, depending on the predator's type and location: (*i*) *A‐call sequences* for aerial predators in the canopy (i.e. raptors); (*ii*) *B‐call sequences* for terrestrial predators on the ground (i.e. felids or tayras); (*iii*) *one A‐call followed by B‐calls* for terrestrial predators in the canopy; and (*iv*) *A‐calls interspersed with B‐calls* for aerial predators on the ground.

Based on these findings, the authors concluded that A‐calls refer to aerial predators, B‐calls to terrestrial predators, and that the sequence structure conveys additional information about the predator's location (Cäsar *et al*., 2012b, 2013). This system was thus presented as a complex interface between semantics, where each call has an independent meaning, and syntax, where the structure of the sequence provides additional meaning (Cäsar & Zuberbühler, 2012) (Table 1).

**Table 1:** Summary of competing theories on titi monkey alarm sequences.

Theory	Proposed meaning of A‐calls	Proposed meaning of B‐calls	Rules of call combinations	Response of receivers	Fit of the theory with the data set presented here
Cäsar *et al*., (2012b, 2013)	‘aerial predator’	‘terrestrial predator’	Combining calls conveys additional information about predator location (e.g. A then B = terrestrial predator in the canopy)	Respond according to predator type and location (e.g. receivers know that the sequence encodes a terrestrial predator in the canopy)	**Partial** – calls or sequences are not context specific
Schlenker *et al*., (2017)	‘serious non‐ground alert’	‘noteworthy event but not a serious non‐ground one’, with meaning enriched by the Informativity Principle	Sequences reflect the evolution of the situation over time (e.g. A then B = a felid in a tree is a serious non‐ground threat, but becomes less serious once deterred by alarm calls). No syntax	Pragmatic inferences based on world knowledge (e.g. receivers infer that a situation evolving from a serious non‐ground threat to a noteworthy event likely reflects a terrestrial predator in a tree)	**High** – explains sequence structure, but not the high variability of sequences within a given context
Variation of Schlenker *et al*. theory (Commier & Berthet, 2019)	‘serious alert’	‘noteworthy event but not a serious one’, with meaning enriched by the Informativity Principle	Sequences reflect the evolution of the situation over time (e.g. A then B = a felid in a tree is a serious threat, but becomes less serious once deterred by alarm calls). No syntax	Pragmatic inferences based on world knowledge (e.g. receivers infer that a situation evolving from a serious threat to a noteworthy event likely reflects a terrestrial predator in a tree)	**High** – explains sequence structure, but not the high variability of sequences within a given context. More parsimonious than Schlenker *et al*. theory.
Present review	High arousal state	Low arousal state	Sequences retrace changes in arousal level over time (e.g. A then B = the caller is surprised, then calms down). No syntax	Pragmatic inferences based on world knowledge (e.g. receivers infer that a caller experiencing high then lower arousal states probably encountered a terrestrial predator in a tree)	**Very high** – accounts for the high variability and the lack of context specificity of the sequences, and explains the sequence structure

Each proposes a different interpretation of the semantic, pragmatic and syntactic properties of the system. The present review suggests the most parsimonious explanation of the available data.

Schlenker *et al*. (2016a, 2017) and Schlenker, Chemla & Zuberbühler (2016b) subsequently conducted formal linguistic analyses on the data set of Cäsar *et al*. (2012b, 2013). They concluded that A‐calls refer to serious non‐ground alerts while B‐calls indicate noteworthy events. The two calls vary greatly in their informativity levels: A‐calls are more informative than B‐calls, in the sense that they provide more accurate information. Because titi monkeys might use the most informative call whenever possible (‘informativity principle’), this results in a pragmatic enrichment for B‐calls: if B‐calls are emitted, listeners can infer that the event is noteworthy, but it is not a non‐ground serious event (otherwise, an A‐call would have been emitted instead). More importantly, Schlenker *et al*. (2017) argued that the sequences are not structured following syntactic rules, but rather that the calls are emitted independently, with the sequences reflecting the evolution of the context over time (Table 1). Specifically, when encountering a raptor in the canopy, titi monkeys would emit only A‐calls, as this represents a serious non‐ground event. By contrast, they would emit only B‐calls when encountering a felid on the ground, since it is a noteworthy event but felids are not the main predators of these primates (Ferrari, 2009). When a raptor is spotted on the ground, titi monkeys would initially emit A‐calls since the raptor is a serious non‐ground threat, but they would switch to B‐calls once they realise the raptor is not in a hunting position and thus, not serious. Similarly, when encountering a terrestrial predator in the canopy, titi monkeys would emit one A‐call, as it is both a dangerous and non‐ground threat (since it is in the canopy). They would then switch to B‐calls because, once the felid has been spotted, it becomes less dangerous. Indeed, felids rely on ambush attacks (Zuberbühler, David & Bshary, 1999), so the production of alarm calls may stop the predator from hunting.

A further refinement of this analysis proposed that A‐calls refer to serious alerts (and not ‘serious non‐ground’ alerts) (Commier & Berthet, 2019): the ‘non‐ground’ component of the meaning was deemed redundant with its ‘serious’ component since all dangerous threats are non‐ground for titi monkeys (Ferrari, 2009). The core assumptions of Schlenker *et al*. (2017) remain intact: titi monkeys switch from A‐calls to B‐calls in both the raptor‐on‐the‐ground and terrestrial‐predator‐in‐the‐canopy situations because they recognise that in both cases, the threat becomes less serious over time (Commier & Berthet, 2019). Under this analysis, the titi monkey alarm system presents an interesting system with an interface between semantics (calls have an independent meaning) and pragmatics (the meaning of B‐calls can be enriched through call competition), but no involvement of syntactic processes.

The question of whether the titi monkey alarm system is syntactic remains unresolved until new data are introduced. To address this, Berthet *et al*. (2018, 2019, 2022) and Narbona Sabaté *et al*. (2022) replicated the experiments of Cäsar *et al*. (2012b, 2013) and conducted additional field experiments to explore whether the titi monkey alarm system involves complex semantic and syntactic capacities.

## III. OUR DATA SET

The data presented here have been published previously (Berthet *et al*., 2018, 2019, 2022; Narbona Sabaté *et al*., 2022) and represent the most comprehensive data set on titi monkey alarm sequences available to date.

Data were collected between 2014 and 2016 at the Reserva Particular do Patrimônio Natural Santuário do Caraça, Minas Gerais, Brazil, from six wild groups of black‐fronted titi monkeys (mean ± SD: 5 ± 0.58 individuals/group) habituated to human presence. The monkeys were presented with four taxidermised predator models: two models of a raptor (*Caracara plancus*), one tayra (*Eira barbara*), and one oncilla (*Leopardus guttulus*). Each predator species was presented once in the canopy and once on the ground to each titi monkey group. We recorded group responses, initially consisting solely of the vocalisations of the first individual to detect the predator (the spotter); if other individuals vocalised, they only began calling after the spotter had produced at least 10 calls.

Consistent with the findings of Cäsar *et al*. (2013), the composition of alarm sequences varied with the type and location of the predator (Berthet *et al*., 2019; Narbona Sabaté *et al*., 2022). Specifically, sequences given in response to taxidermised raptors were primarily composed of A‐calls, although B‐calls were also emitted when the raptor model was on the ground. Sequences given in response to taxidermised terrestrial predators were mainly composed of B‐calls, although A‐calls were also emitted when the terrestrial predator model was in the canopy (Berthet *et al*., 2019) (Table 2).

**Table 2:** Vocal sequences (30 first calls only) emitted by six groups of titi monkeys (A, D, M, P, R and S) when exposed to taxidermised predators (top panel) and predator vocalisations (bottom panel).

Experiment	Predator	Location	Group	1	2	3	4	5	6	7	8	9	10	11	12	13	14	15	16	17	18	19	20	21	22	23	24	25	26	27	28	29	30
Predator presentations	Raptor (caracara)	Canopy	A	A																													
			M	A	A	A	A	A	A	A	A	A	A	A	A	A	A	A	A	A	A	A	A	A	A	A	A	A	A	A	A	A	A
			P	A	A	A	A	A	A	O	A	O	O	A	A	O	A	O	O	B	A	A	A	B	A	B	O	B	A	A	A	A	A
			R	A	A	A	A	A	A	A	A	A	A	A	A	A	A	B	B	A	A	A	A	A	A	A	A	A	A	A	A	A	B
			S	A	A	A	A	A	A	A	A	A	A	A	A	A	A	A	A	O	A	A	A	B	B	B	A	B	B	B	B	B	B
		Ground	A	B																													
			D	A	A	A	A	A	A	A	A	B	A	A	A	A	A	A	A	A	A	A	A	A	B	B	B	B	B	A	B	B	B
			M	A	A	A	A	A	A	A	A	A	A	A	A	A	A	B	O	A	B	B	B	B	A	B	B	A	A	A	A	B	O
			P	A	A	B	A	A	B	A	O	B	A	A	B	B	B	B	B	B	B	B	B	B	B	O	O	A	B	B	B	B	B
			R	B																													
			S	A	A	A	A	A	A	A	A	A	A	A	A	A	A	A	A	A	A	A	B	A	A	A	A	A	A	A	A	A	AS
	Felid (oncilla)	Canopy	A	B	B	B	B	B	B	B	B	B	B	B	B	B	B	B	B	B	B	B	B	B	B	B	B	B	B	B	B	B	B
			D	B	B	B	B	A	B	B	B	B	B	B	B	B	B	B	B	B	B	B	B	B	B	B	B	B	B	B	B	B	B
			M	B	B	B	B	B	B	B	B	B	B	B	B	B	B	B	B	B	B	B	B	B	B	B	B	B	B	B	B	B	B
			P	B	B	B	B	B	B	B	B	B	B	B	B	B	B	B	B	B	B	B	O	B	B	B	B	B	B	B	B	B	B
			R	B	A	A	B	B	B	B	A	B	B	B	B	B	B	B	B	B	B	B	B	B	B	B	B	B	B	B	B	B	B
			S	B	B	B	B	B	B	B	B	B	B	B	B	B	B	B	B	B	B	B	B	B	A	B	B	B	B	B	B	A	B
		Ground	A	B	B	B	B	B	B	B	B	B	B	B	B	B	B	B	B	B	B	B	B	B	B	B	B	B	B	B	B	B	B
			D	B	B	B	B	B	B	B	B	B	B	B	B	B	B	B	B	B	B	B	B	B	B	B	B	B	B	B	B	B	B
			M	B	B	B	B	B	B	B	B	B	B	B	B	B	B	B	B	B	B	B	B	B	B	B	B	B	B	B	B	B	B
			P	A	B	B	B	B	B	B	B	B	B	B	B	B	B	B	B	B	B	B	B	B	B	B	B	B	B	B	B	B	B
			R	B	B	B	B	B	B	B	B	B	B	B	B	B	B	B	B	B	B	B	B	B	B	B	B	B	B	B	B	B	B
			S	B	B	B	B	B	B	B	B	B	B	B	B	B	B	B	B	B	B	B	B	B	B	B	B	B	B	B	B	B	B
	Tayra	Canopy	A	A	B	B	B	B	B	B	B	B	B	B	B	B	B	B	B	B	B	B	B	B	B	B	B	B	B	B	B	B	B
			D	B	B	B	B	B	B	B	B	B	B	B	B	B	B	B	B	B	B	B	B	B	B	B	B	B	B	B	B	B	B
			M	B	B	B	B	B	B	B	B	B	B	B	B	B	B	B	B	B	B	B	B	B	B	B	B	B	B	B	B	B	B
			P	B	B	B	B	B	B	B	B	B	B	B	B	B	B	B	B	B	B	B	B	B	B	B	B	B	B	B	B	O	B
			R	A	B	B	B	B	B	B	B	B	B	B	B	B	B	B	O	B	B	B	B	B	B	B	B	B	B	B	B	B	B
			S	B	B	B	B	B	B	B	B	B	A	B	O	B	B	B	B	B	A	A	B	B	B	B	B	B	B	B	B	B	B
		Ground	A	B	B	B	B	B	B	B	B	B	B	B	B	B	B	B	B	B	B	B	B	B	B	B	B	B	B	B	B	B	B
			D	B	B	B	B	B	B	B	B	B	B	B	B	B	B	B	B	B	B	B	B	B	B	B	B	B	B	B	B	B	B
			M	B	B	B	B	B	B	B	B	B	B	B	B	B	B	B	B	B	B	B	B	B	B	B	B	O	B	B	B	B	B
			P	B	B	B	B	B	B	B	B	B	B	B	B	B	B	B	B	B	B	B	B	B	B	B	B	B	B	B	B	B	B
			R	B	B	B	B	B	B	B	B	B	B	B	B	B	O	B	B	B	B	B	B	B	B	B	B	B	B	B	B	B	B
			S	B	B	B	B	B	B	B	B	B	B	B	B	B	B	B	B	B	B	B	B	B	B	B	B	B	B	B	B	B	B
Predator playbacks	Raptor (caracara)	Canopy	A	A	A	A	A																										
			D	A	A	A	A	A	A	A	O	A																					
			M	A	A	A	A	O																									
			P	A																													
			R	A	A	A	A	A	A	A	A	A	A																				
			S	A	A	O																											
		Ground	A	A																													
			D	A	A	A	A	A																									
			M																														
			P	O																													
			R	A	A	A																											
			S	A	O	O	O	A	O																								
	Felid (ocelot)	Canopy	A	B	B	B	A	B	O	B																							
			D	A	A	A	A	A	B	A	A	B	B	B	B	B	B	B	B	B	B	B	B	B	B	B	B	B	B	B	B	B	B
			M																														
			P	B	B	O	A																										
			R	A	O	A	A																										
			S																														
		Ground	A	B	B	B	B	O																									
			D	A	A	A	B	B	A	B	B	B	B	B	B	B	B	B	B	A	B	B	B	B	A	A	A	B	A	A	A	B	A
			M	A	A	A																											
			P																														
			R	A	A	A	A																										
			S																														

Each row represents the vocal reaction of one group of monkeys in one condition, call types are colour‐coded: A‐call in pink; B‐call in green; other calls or unknown calls (O) in orange.

To complement the predator presentation experiments, predator playback experiments were conducted where each group of titi monkeys was exposed to vocalisations of a raptor (*Caracara plancus*) and a felid (*Leopardus pardalis*), broadcast from either the ground or the canopy. Given the close spatial cohesion of titi monkey groups, all individuals were simultaneously exposed to the playback stimuli; vocal responses were therefore recorded at the group level. Surprisingly, the results from these playbacks differed from the model presentations. First, alarm sequences of titi monkeys did not vary substantially with the location of the loudspeaker when exposed to predator calls. Second, vocal responses were inconsistent between the playback of predator calls and model presentations. Indeed, titi monkeys emitted similar alarm sequences when exposed to taxidermised raptors and raptor calls (Berthet *et al*., 2022) (Table 2). However, when exposed to felid calls, titi monkeys either did not vocalise or emitted sequences similar to those produced for taxidermised raptors and raptor calls (i.e. sequences starting with an A‐call and mainly composed of A‐calls) (Berthet *et al*., 2022) (Table 2).

Playback experiments of conspecific alarm sequences were also conducted, where the gaze reactions of listeners were recorded. These experiments revealed that listeners look more upward when hearing sequences elicited by aerial models or models in the canopy, and more towards the loudspeaker when hearing sequences elicited by terrestrial models (Berthet *et al*., 2019). These gaze reactions appeared to depend on the proportion of BB‐grams (i.e. B‐call pairs) in the sequence: the more BB‐grams, the less the titi monkeys looked upward and the more they looked toward the loudspeaker (Berthet *et al*., 2019). Finally, additional field observations showed that B‐calls were also produced in non‐predatory contexts, for example when titi monkeys encountered a non‐predatory animal or when they moved towards the ground to feed (Cäsar *et al*., 2012a; Berthet *et al*., 2018).

## IV. THE SYNTAX OF THE ALARM SEQUENCES

In this review, we will evaluate whether the alarm system of titi monkeys operates under complex semantic and syntactic rules, as suggested previously (Cäsar, 2011; Cäsar *et al*., 2012b, 2013), or whether it can be explained by simpler mechanisms. To address this question, we will adopt the framework developed by Schlenker *et al*. (2016a, 2023, 2024) for evaluating compositionality in animal communication. Schlenker *et al*. approach involves comparing three competing hypotheses.

The first hypothesis (*rich hypothesis*) posits that titi monkey alarm sequences are genuinely compositional. According to this hypothesis, A‐ and B‐calls are systematically combined using syntactic rules, with each call carrying meaning. The overall sequence's meaning emerges from both individual calls and the way they are combined (Berthet *et al*., 2023). This would be similar to human compositional phrases, like ‘the blue dress’.

The second hypothesis (*deflationary hypothesis 1*) suggests that titi monkey alarm sequences are ‘only one expression’, i.e. they are in fact a single complex vocalisation rather than a combination of shorter calls. Under this hypothesis, a sequence is in fact one single standalone call, which bears meaning, but only superficially looks like a succession of A‐ and B‐calls, hence making syntactic rules unnecessary. This would be similar to how certain words resemble combinations of meaningful words, while in fact they are not – e.g. ‘butterfly’ looks like the combination of ‘butter’ and ‘fly’, although it is not.

The third hypothesis (*deflationary hypothesis 2*) proposes that the alarm sequences consist of independent calls produced in close succession, but that do not form a complex combination: they are ‘separate utterances’. Here, the meaning of the sequences arises from the mere conjunction of the individual call meanings, without requiring syntactic rules. This would be similar to people playing a ‘hot and cold game’, where one can say long sentences like ‘cold, cold, cold, hotter, hotter, colder, hotter’ to help someone else retrieve an item: the sentence does not follow a syntactic rule.

We now assess whether the data set allows us to reject the two deflationary hypotheses. If both deflationary hypotheses can be ruled out, we can confidently conclude that titi monkeys possess a compositional alarm system. However, if at least one remains plausible, we cannot assert that titi monkey alarm sequences are truly compositional.

### (1) Deflationary hypothesis 1: alarm sequences are only one expression

This hypothesis proposes that what appears to be a sequence is actually a single complex vocalisation rather than a combination of shorter calls. While this hypothesis may be relevant when analysing short, two‐call combinations (e.g. Schlenker *et al*., 2023, 2024), it becomes untenable for longer sequences like those of titi monkeys. Indeed, titi monkey alarm sequences can last up to 2 h and include thousands of calls, making it highly unlikely that a vocalisation, even complex, would extend over such a duration.

Moreover, titi monkeys produce calls at a relatively slow pace. For example, the first 10 calls of a sequence are separated by an average of 1.98 s of silence (see Supplementary Materials in Berthet *et al*., 2019). Some sequences remain identical for the first 16 calls and diverge only at the 17th call, after more than 30 s. If a sequence is only one vocalisation, monkeys would have to wait a considerable amount of time before distinguishing the meaning of the expression they are hearing (and thus, what information to extract), a strategy that seems maladaptive in predator‐related contexts where rapid responses are crucial.

Furthermore, titi monkey sequences present extremely high variability despite being elicited in a limited range of situations. Analysing just the first 30 calls, we observe 40 distinct sequences across 59 experimental trials (Table 2). If each sequence was only one expression, this would imply an extensive repertoire that individuals would need to learn to communicate effectively about predation events – an unlikely scenario. This is particularly evident when considering models emphasising the computational advantages of combinatorial systems, which allow for the generation of new meanings without learning entirely new calls (Nowak Plotkin & Jansen, 2000).

Finally, playback experiments show that upon hearing conspecific alarm sequences, titi monkeys respond immediately by looking up (A‐calls) or towards the loudspeaker (B‐calls) (latency of less than 0.5 s; Cäsar *et al*., 2012b). This rapid reaction suggests that they do not need to process the entire sequence to react appropriately, reinforcing the idea that individual calls are interpreted independently.

Taken together, these arguments strongly suggest that titi monkey alarm sequences are not an ‘only one expression’, but are instead composed of independent, meaningful calls. Therefore, we can confidently reject deflationary hypothesis 1.

### (2) Deflationary hypothesis 2: alarm sequences are composed of separate calls

An alternative hypothesis suggests that titi monkey alarm sequences consist of meaningful calls produced in close succession but without an underlying syntactic rule. In this case, each call is produced independently, and receivers extract meaning by summing the information conveyed by the independent calls.

This hypothesis is difficult to refute. Indeed, as mentioned previously, the high variability in titi monkey alarm sequences suggests either the absence of a syntactic rule or a highly complex rule not easily identifiable. It seems more likely that each call is emitted independently. Additionally, critical information sometimes appears late in the sequence: if a syntactic rule governed these sequences, receivers would occasionally need to wait over 30 s to extract the relevant information, which, again, would be maladaptive in high‐risk situations. Instead, it is more likely that receivers process each call sequentially, extracting meaning in real time rather than waiting for the full sequence. This interpretation is further supported by the observation that titi monkeys react after hearing only one single call, reinforcing the hypothesis that they do not process alarm sequences as syntactic structures but rather as a sum of independent calls. Given our data set, we cannot refute deflationary hypothesis 2.

Therefore, for the remainder of this work, we will assume that titi monkey alarm sequences are explained by deflationary hypothesis 2, i.e. sequences are composed of independently produced calls without any syntactic rule governing their association. Under this hypothesis, each call is meaningful, and the overall information encoded at the sequence level is simply the addition of the successive meanings of individual calls. In the next section, we will investigate what specific information these calls encode.

## V. THE SEMANTICS OF A‐ AND B‐CALLS

The semantics of animal signals can be divided into four main types: (*i*) meanings that denote external events (e.g. ‘there is a leopard’); (*ii*) meanings that describe the receiver's behaviour (e.g. ‘run away’); (*iii*) meanings that describe the signaller's future behaviour (e.g. ‘I will run away’); and (*iv*) meanings that express the signaller's internal state (e.g. ‘I am afraid’) (Berthet *et al*., 2022; Berthet, Surbeck & Townsend, 2025).

The first studies on the titi monkey alarm call system suggested that both A‐ and B‐calls, and by extension, the sequences, encode information related to external events: the predator type and its location (Cäsar *et al*., 2012b, 2013; Berthet *et al*., 2019). However, this interpretation is challenged by the complexity of the data set (Schlenker *et al*., 2017). A‐calls are given in all predatory situations (Table 2), regardless of whether the predator is seen or heard, terrestrial or aerial, on the ground or in the canopy. Similarly, B‐calls are produced in all predatory situations except in response to the call of a raptor (Table 2, playback of caracara). Moreover, B‐calls also occur in non‐predatory contexts, such as when encountering a deer or feeding near the ground (Cäsar *et al*., 2012a; Berthet *et al*., 2018). Given their occurrence across a wide range of situations that, from a human perspective, appear only loosely related, it is difficult to assign a strictly referential meaning to A‐ and B‐calls based on predator type, location, or mode of detection.

This challenge becomes even more complex when considering the high variability of titi monkey alarm sequences. While some patterns emerge (e.g. caracaras often elicit sequences consisting only of A‐calls or sequences with A‐calls preceding B‐calls), these patterns are not strictly context dependent: the same type of sequence can be given in different situations, and no specific situation consistently elicits the same sequence (Table 2). More surprisingly, sequences given in responses to terrestrial predator vocalisations resemble those given in response to raptors more than those given to models of terrestrial predators. Together, these findings suggest that titi monkey alarm calls do not refer to external events but rather encode information that varies across trials.

Following this reasoning, we hypothesise that the meaning of the calls reflects an internal state of the caller. While alarm calls can encode relatively stable individual features such as age, sex or rank (e.g. Price *et al*., 2008; Narbona Sabaté *et al*., 2022), these are unlikely to be the primary information conveyed in titi monkey alarm calls, as alarm calling occurs in highly dangerous situations where rapid and adaptive responses from receivers are critical. While, in some species with strong intra‐group competition, individuals may benefit from signalling their propensity to protect the group against threats (e.g. Mehon & Stephan, 2021), this is unlikely the case in titi monkey as groups have little to no hierarchy (Bicca‐Marques & Heymann, 2013), and individuals do not need to prove their usefulness to remain in the group. Therefore, the most relevant information titi monkeys might communicate in a predatory situation is their own perception of the level of urgency – or, in other words, their arousal level. Similar systems where different call types encode different emotional states have been described in several other species (see Engesser & Townsend, 2019), ranging from primates (Price *et al*., 2015; Liao *et al*., 2018) to meerkats (*Suricata suricatta*) (Manser, 2001) and rock cavies (*Kerodon rupestris*) (Almeida *et al*., 2025). Here, we thus hypothesise that titi monkeys emit calls that encode their arousal state at the time of emission of the call, and that the whole sequence retraces changes in arousal level over time.

Supporting this interpretation, A‐calls are more commonly produced in response to raptors and non‐visible predators (i.e. playback of predator sounds). Given that raptors are considered the most serious threat to small American monkeys (Ferrari, 2009), A‐calls likely indicate higher arousal levels. By contrast, B‐calls are more frequently given in response to terrestrial predators, which, while dangerous, are less agile in the trees and typically target larger prey (Ferrari, 2009). B‐calls are also emitted in noteworthy non‐predatory events that require vigilance but do not pose immediate threats. These observations suggest that B‐calls may indicate lower arousal levels. Strikingly, when both A‐ and B‐calls occur within the same sequence, A‐calls tend to occur earlier in the sequence, followed by B‐calls (12/18 sequences). This further supports the hypothesis that A‐calls indicate higher arousal levels: upon initially detecting a potential threat, a titi monkey is likely to experience a peak in arousal, which may decline over time as the situation becomes clearer (e.g. the model remains motionless and in an unusual place, or the group is now fully informed).

Based on these observations, we propose that A‐calls reflect higher arousal at the time of production, B‐calls reflect lower arousal, and that a sequence of calls as a whole represents fluctuations in arousal over time. Below, we examine whether this hypothesis aligns with the data set (see Table 2).

For *raptor models*, since raptors are the most dangerous threat to American primates, titi monkeys exhibit a strong arousal response when spotting a caracara, emitting A‐calls. Over time, if the raptor remains motionless or the group becomes fully informed, arousal may decrease, leading to a shift from A‐calls to B‐calls. Interestingly, one group of monkeys (Group A) displayed a weaker response than others, producing only one A‐call in the canopy condition and one B‐call in the ground condition. This could indicate differences in past experiences with raptors or a greater ability to recognise the model as non‐threatening.

For *oncilla and tayra models*, while terrestrial predators require vigilance, they pose a less immediate threat to titi monkeys, as they are less agile in trees and typically target larger prey (Ferrari, 2009). Accordingly, titi monkeys primarily respond with B‐calls, indicating moderate arousal. Occasionally, an A‐call appears at the start of a sequence, particularly when the predator is in the canopy. This initial burst of A‐calling may reflect surprise – an adaptive ‘better safe than sorry’ strategy, where titi monkeys initially react as if the predator could be a raptor before confirming its identity and switching to B‐calls. In rare cases, A‐calls occur later in the sequence, potentially reflecting the response of a newly alerted individual or a shift in arousal due to unrecorded contextual factors, such as approaching the model.

For *caracara calls*, because raptors pose an extreme threat, titi monkeys react with high arousal, producing A‐calls. Since the caller cannot locate the raptor, arousal remains high and no transition to B‐calls occurs.

Responses to *ocelot calls* are more varied, suggesting that titi monkeys may not recognise them as belonging to a predator. In some cases (e.g. Group A), reactions resemble those given to terrestrial models, with B‐calls indicating moderate arousal. In others, sequences contain mostly or exclusively A‐calls, or monkeys fail to respond vocally at all. This inconsistency suggests that titi monkeys may not have learned to associate these vocalisations with felids. Many primate species fail to recognise the vocalisations of their predators (e.g. Hettena, Munoz & Blumstein, 2014; Deppe, 2020), likely due to limited exposure – felids are solitary, low‐density predators that rarely vocalise, especially when hunting (Hettena *et al*., 2014). In cases where titi monkeys respond with A‐calls, they may be exhibiting a general response to unfamiliar, stressful sounds, following a precautionary ‘better safe than sorry’ strategy. When no calls are given, the sound may not be perceived as threatening.

Descending from the canopy increases vulnerability to predation (Mourthé *et al*., 2007), particularly in dense forest habitats where visibility is low. However, because no predator is present (the individual would not descend otherwise), the threat remains moderate. This likely results in mid‐level arousal, leading to the production of B‐calls during descent.

Taken together, these observations support the hypothesis that titi monkey alarm calls reflect the caller's arousal level at the time of emission, with sequences tracking changes in arousal over time.

## VI. THE PRAGMATICS OF THE ALARM SEQUENCES

We have established that alarm calls likely reflect the caller's arousal level at the time of emission of the call (deflationary hypothesis 2). However, strikingly, titi monkeys can still extract relevant information about the predator and its location from alarm sequences. Playback experiments show that receivers adjust their gaze direction based on the composition of the sequence: when hearing sequences composed of more BB‐grams, i.e. sequences given to terrestrial models or models placed on the ground, titi monkeys look more towards the loudspeaker and less upwards (Berthet *et al*., 2019).

Whilst titi monkeys seem to extract contextual information from alarm sequences, they do not rely on semantics to do so if, as we posit, the calls themselves encode the caller's arousal level rather than direct information about the predator's type or location. Instead, they likely use pragmatics, drawing on their knowledge of the world to infer the situation eliciting the caller's reaction. For instance, titi monkeys know that raptors are the most dangerous predators and typically attack from above. When hearing A‐calls, which signal high arousal in the caller, receivers may infer the presence of a severe threat – most likely a raptor – and immediately look upwards to scan for danger. If the caller subsequently switches to B‐calls, the receiver may interpret this as an indication that the threat is no longer immediate, prompting them to shift their attention towards the caller for further cues and adjust their response accordingly. Notably, it is the emission of BB‐grams, i.e. pairs of B‐calls, rather than isolated B‐calls within the sequence, that significantly influences the behaviour of the listener (Berthet *et al*., 2019). This suggests that the receiver monitors the arousal level of the caller over a longer period of time before concluding that the threat really is no longer serious (again, a ‘better safe than sorry’ strategy): while isolated B‐calls would merely indicate transient lower arousal states, BB‐grams probably reflect a sustained state of low arousal. Conversely, if the sequence consists only of B‐calls, the receiver may infer that the caller is experiencing mild arousal and looks towards the caller to investigate further the source of the disturbance (e.g. the caller may be close to the ground, or there may be a terrestrial predator).

Our hypotheses need observational or experimental validation. Future work can, for example, combine acoustic recordings with physiological monitoring, such as heart rate or nasal skin temperature (Dezecache *et al*., 2017; Liao *et al*., 2018), to quantify arousal levels at the onset of calls. Another option would be to assess whether individual personality traits, such as reactivity or emotionality (Suomi, Chaffin & Higley, 2011), influence the relative production of A‐ and B‐calls.

## VII. IMPLICATIONS FOR OUR UNDERSTANDING OF LANGUAGE EVOLUTION

In this work, we investigated whether the titi monkey alarm system exhibits complex semantics and syntactic properties, as first proposed by Cäsar *et al*. (2012b, 2013). Using a linguistic analysis, our study shows that alarm sequences are composed of independently produced calls with no syntactic rule governing their combination. Each call is meaningful, and the meaning of the sequence is not compositional but purely additive, arising from the successive accumulation of individual call meanings. This conclusion is similar to that of Schlenker *et al*. (2017), although our enriched data set leads to an important reinterpretation. In the conclusions drawn by Schlenker *et al*. (2017), A‐ and B‐calls semantically designate external referents, namely the type and level of urgency of the threat. By contrast, we argue that these calls may instead reflect the caller's internal state, with no evidence for referentiality. In this sense, our conclusions are more parsimonious (Table 1).

Because compositional syntax is considered a hallmark of human language, many recent studies have focused on identifying syntax‐like properties in non‐human communication to trace its evolutionary origins. Indeed, evidence of compositional‐like structures has been reported in the vocal systems of great apes and birds (Engesser *et al*., 2016; Suzuki, Wheatcroft & Griesser, 2016; Suzuki & Matsumoto, 2022; Leroux *et al*., 2023; Berthet *et al*., 2025). Against this background, the absence of evidence for compositional syntax in titi monkeys could suggest that their alarm system – and maybe, their communication system more broadly – offers little insight into the evolution of complex communication. However, we argue the opposite: although titi monkey alarm call sequences appear to lack syntactic structuring, they nonetheless seem to be informative, and as such represent a powerful model for understanding the evolution of other key features of human language, such as pragmatics. In titi monkeys, callers produce relatively simple signals shaped by immediate emotional arousal, whereas receivers enrich these signals pragmatically by integrating background knowledge and therefore, engage in more complex inferential processes. This striking asymmetry suggests that cognitively sophisticated processes can emerge not only from the signal production perspective, but also from its interpretation.

Historically, emotional communication was dismissed as primitive and largely excluded from debates on language origins (Foolen, 2022). Philosophers such as de Condillac (1746) considered that language emerged when humans managed to bring their natural expression under voluntary control. Wundt (1900, pp. 319–322) saw interjections as ‘linguistic remains’ of animal emotional expression, and attributed the decline of interjections in modern culture to ‘the tempered expression of emotion as required by civilisation’. Similarly, Sapir (1921, p. 8) described language as a ‘system of voluntarily produced symbols’, relegating emotion to a secondary role. Although linguists began to shift away from this perspective by studying the relation between language and emotion in the 20th century (Foolen, 2022), this framing deeply influenced the field of animal communication. Because language was primarily considered a referential system, emotion‐related signals in animals were often considered reflexive, automatic, and devoid of intentionality (Townsend *et al*., 2017; Heesen *et al*., 2021). Consequently, research on the evolution of language has focused heavily on detecting referential, ‘functionally referential’, or emotion‐decoupled signals (Seyfarth *et al*., 1980b; Crockford *et al*., 2012; Crockford, Wittig & Zuberbühler, 2017; Taylor *et al*., 2022, 2023).

Yet this perspective has increasingly been challenged. Seyfarth & Cheney (2003) argued that the dichotomy between referential and emotional (or ‘affective’) communication is misleading, since affective signals can still function referentially when the underlying emotional state reliably correlates with specific external events, allowing the listeners to extract information from them. Similarly, the assumption that emotional communication is necessarily non‐intentional has been weakened. Human interjections (e.g. ‘Damn!’), which convey information about emotional states, are clearly used intentionally (Wharton, 2009); in animals, signals produced in high‐arousal contexts can also be under voluntary control or intentional (Slocombe & Zuberbühler, 2007; Schel *et al*., 2013, 2013). This led Heesen *et al*. (2021) to propose a framework where intentionality and emotional valence of the signal are considered as two independent dimensions of a signal.

Finally, Wheeler & Fischer (2012) suggested that focusing exclusively on referential signals may obscure the detection of precursors of language. Referential signals can often be processed in a simple, context‐free manner, whereas responding to more ambiguous, less context‐specific signals requires sophisticated inferential abilities, including pragmatic reasoning. By this account, pragmatic communication may offer a better model for the origins of language than strictly referential ones.

In this light, the titi monkey system becomes especially valuable. Even if it remains unclear whether alarm calls are produced intentionally, their interpretation clearly engages complex inferential processes. This challenges the traditional view of emotional communication as cognitively trivial, showing instead that it can drive pragmatic enrichment in receivers [see Arnold & Bar‐On (2020) for a similar argument].

Our findings suggest that emotion‐based communication, far from being primitive, may provide crucial insights into the cognitive foundations of human language. With this review, we encourage researchers to re‐examine the role of emotional communication in the emergence of linguistic‐like capacities in non‐human species.

## VIII. CONCLUSIONS

(1) Titi monkey alarm calls most plausibly convey the caller's arousal state at the time of emission.
(2) Titi monkey alarm sequences likely reflect fluctuations of this arousal state over time without requiring syntactic rules.
(3) Receivers use pragmatics to infer the most adaptive response to these calls.
(4) Further experimental and observational work will be necessary to validate these findings.
(5) Ultimately, this review advocates for the integration of non‐human emotional communication into research on language evolution, highlighting its relevance for understanding the origins of human language.

## Data Availability

Data sharing not applicable to this article as no datasets were generated or analysed during the current study.
